# Barriers to Family Building Among Physicians and Medical Students

**DOI:** 10.1001/jamanetworkopen.2023.49937

**Published:** 2023-12-28

**Authors:** Zoe King, Qiang Zhang, Jane W. Liang, Morgan S. Levy, Torie C. Plowden, Roohi Jeelani, Ariela L. Marshall, Rebecca Barnett, Alberto J. Caban-Martinez, Alyssa Brown, Claudia M. Mueller, Cati Brown-Johnson, Arghavan Salles

**Affiliations:** 1 Division of Primary Care and Population Health, Department of Medicine, Stanford University School of Medicine, Palo Alto, California; 2 Stanford Prevention Research Center, Stanford University School of Medicine, Palo Alto, California; 3 University of California, Los Angeles David Geffen School of Medicine, Los Angeles; 4 Quantitative Sciences Unit, Department of Medicine, Stanford University School of Medicine, Palo Alto, California; 5 University of Miami Miller School of Medicine, Miami, Florida; 6 Department of Public Health Sciences, University of Miami Miller School of Medicine, Miami, Florida; 7 Department of Gynecologic Surgery and Obstetrics, Walter Reed National Military Medical Center, Bethesda, Maryland; 8 Department of OB/GYN, Division of Reproductive Endocrinology and Infertility Wayne State University School of Medicine, Detroit, Michigan; 9 Kindbody Fertility Clinic, Chicago, Illinois; 10 Department of Medicine, Division of Hematology, Oncology, and Transplantation, University of Minnesota, Minneapolis; 11 Department of Surgery, Northwestern University Feinberg School of Medicine, Chicago, Illinois; 12 Department of Surgery, Stanford University School of Medicine, Palo Alto, California; 13 Department of Medicine, Stanford University School of Medicine, Palo Alto, California; 14 Clayman Institute for Gender Research, Stanford University School of Medicine, Palo Alto, California

## Abstract

**Importance:**

Physicians and medical students who desire to build families face significant barriers due to the structure and culture of medicine.

**Objective:**

To understand the barriers and facilitators to family building for all people in medicine—not only individuals who can become pregnant—through an open-ended, qualitative analysis of survey responses.

**Design, Setting, and Participants:**

This qualitative study used a survey conducted in April and May 2021 with a broad sample of physicians and medical students. Participants were recruited through social media, targeting physician and medical student communities. Physicians (residents, fellows, and physicians in independent practice) and medical students of all gender identities and sexual orientations were included. Informed by a postpositivist approach, coding reliability thematic analysis was performed on 3 open-ended survey questions on family-building experiences (what they would do differently, what advice they have for others, and anything else they wished to share).

**Main Outcomes and Measures:**

Identified themes were mapped to the social-ecological model, a model used in public health to examine how a spectrum of factors is associated with health outcomes.

**Results:**

A total of 2025 people (1860 [92%] women; 299 [15%] Asian, 151 [8%] Black, and 1303 [64%] White; 1730 [85%] heterosexual; and 1200 [59%] physicians who had completed training) responded to at least 1 of 3 open-ended questions. Themes mapped to social-ecological model levels included: (1) cultural, eg, medical training being at odds with family building; (2) organizational, eg, lack of institutional support for the range of family-building routes; (3) interpersonal, eg, impact of social support on family building; and (4) individual, eg, socioeconomic status and other individual factors that facilitate or inhibit family building. Recommendations to improve family-building experiences include implementing family-building curricula at medical schools, providing adequate parental leave for all physicians and medical students who become parents, and providing insurance coverage for all family-building routes.

**Conclusions and Relevance:**

In this qualitative study of physicians and medical students, self-reported barriers to family building were identified at each level of the social-ecological model. Addressing these barriers is critical to creating a more equitable family-building environment for physicians and medical students.

## Key Points

**Question:**

What barriers do physicians and medical students face when building their families?

**Findings:**

In a qualitative analysis of 3 open-ended survey questions in a sample of 2025 physicians and medical students, barriers and facilitators to family building were identified and mapped to the social-ecological model (cultural, organizational, interpersonal, and individual). Recommendations included implementing family-building curricula at medical schools, providing adequate parental leave for all physicians, and providing insurance coverage for all family-building routes.

**Meaning:**

In this study, physicians and medical students reported that cultural, organizational, interpersonal, and individual factors created barriers to family building.

## Teaser

This qualitative study examines perceptions of barriers and facilitators to family building among physicians and medical students of different gender identities and sexual orientations.

## Introduction

Although not all physicians and medical students want or are able to have children, those who desire to build families face barriers in the structure and culture of medicine.^1^ Physicians and medical students of all genders and sexual orientations may desire to build families, including through assisted reproductive technologies (ART), fostering, and adoption, among other pathways. In addition, as more women join the physician workforce,^2,3^ more people are becoming pregnant early in their careers, often without adequate support.

Previous studies demonstrated that many women physicians delay childbearing until they finish training.^4,5^ For many physicians, residency overlaps with peak fertility,^6,7,8^ and delaying childbearing until after training is one factor that can contribute to infertility or unfulfilled reproductive goals. It is estimated that one-quarter of women physicians will experience infertility,^7^ which is up to double the rate of the general US population.^9,10^ Few medical schools, residencies, and practices provide adequate education on reproductive life planning, thereby inadequately preparing physicians for this aspect of their lives.^11^ One study found that when reflecting on their family-building experiences, more than a quarter of women physicians would have attempted to build their families earlier.^7^

Certainly family building can be challenging in many occupations; however, the overlap between training and childbearing years combined with the high degree of stigmatization of childbearing within medicine represent somewhat unique challenges. To understand the barriers and facilitators to family building for all people in medicine, we asked a broad sample of physicians and medical students about their experiences and what they would do differently. We use the term *family building* to describe the actions taken by individuals to have children,^12^ but we recognize that having children is not the only way to build a family and that family building is not a priority shared by all people.

## Methods

### Participants and Survey Design

As discussed in prior publications,^13,14,15^ we recruited a convenience sample of physicians (residents, fellows, and physicians in independent practice) and medical students through social media (Twitter, Instagram, Facebook, LinkedIn) in April and May 2021. After providing written informed consent, participants reported demographic, work-related, and family-building journey characteristics. For this study, we analyzed responses to 3 open-ended questions (Box). The study was approved by the University of Miami institutional review board. We followed the Consolidated Criteria for Reporting Qualitative Research (COREQ) reporting guideline elements that were relevant to a qualitative survey study.^16^

Box. **Open-Ended Survey Questions in the Qualitative Analysis**

Q1. What would you do differently, if anything, with regard to family planning knowing what you know now?
Q2. What advice do you have for current trainees who want to have a family?
Q3. Is there anything else we should know about how your career has impacted your family planning?

### Quality and Reflexivity Statement

We report aspects of our identity that may influence the study. Authors identify as women and men who are physicians, medical students, and researchers with specialized experience in qualitative and/or quantitative research. Some authors identify as lesbian, gay, bisexual, transgender, queer (or questioning), intersex, asexual (or allies), and other sexual orientations and gender identities (LGBTQIA+), some have experienced infertility, and some have clinical expertise in reproductive endocrinology and infertility.

### Statistical Analysis

We used a coding reliability thematic approach informed by a postpositivist paradigm to inductively identify themes.^17,18^ Two coders (Z.K. and Q.Z.) read through responses (varying from 1 word to several paragraphs) and confirmed data saturation. Coders completed line-by-line coding, labeling segments of text based on their content, for 200 responses (10% of the data set) to codevelop a codebook draft. Using consensus coding approaches, coders applied the draft codebook to another 300 responses (15% of the data set) and iteratively revised the codebook through team meetings (including A.S. and C.B.-J.). Coders independently coded the full data set using NVivo (QSR International), with substantial interrater reliability (Cohen κ > 0.8). After coding, coders tied responses to self-reported demographic characteristics (career stage, race and ethnicity, sexual orientation, and gender). As the survey was anonymous, we could not follow-up with respondents to validate the data. Race and ethnicity data were collected because they are important aspects of identity that may influence one’s life experiences.

In a subsequent analytic step, we applied the social-ecological model (SEM) to our themes.^19,20^ In multiple team conversations, we defined our interpretation of SEM levels for this study and organized themes according to SEM levels. We chose the SEM as an appropriate model, as it examines how a spectrum of factors is associated with health outcomes, often in preparation for multilevel intervention, a potential end goal of this study.^20,21,22^ SEMs are commonly used in public health, including by the World Health Organization and the Centers for Disease Control and Prevention.^23,24,25^ Based on similar literature, we defined 4 SEM levels: cultural (eg, societal values, discrimination); organizational (eg, policies); interpersonal (eg, social support); and individual (eg, demographics).^26,27,28^

To assess differences between responders and nonresponders to the open-ended questions, we ran a logistic regression model with all demographic variables included as covariates. Missing values were treated as a separate category for categorical variables and were omitted for continuous variables. For demographic variables of primary interest (gender, sexual orientation, and race and ethnicity), we examined all pairwise contrasts, using the Benjamini-Hochberg adjustment for multiple comparisons. Coefficient *P* ≤ .05 was considered statistically significant. We performed all analyses using R version 4.2.1 (R Project for Statistical Computing).

## Results

### Respondent Demographics

Of the 3802 participants who began the survey, 2025 individuals (53%) answered at least 1 of 3 open-ended questions; responders were 1860 (92%) women; 299 (15%) Asian, 151 (8%) Black, and 1303 (64%) White; 1730 (85%) heterosexual; and 1200 (59%) physicians in independent practice. Compared with all participants, those in this subset were more likely to be Black than any of the other racial and ethnic groups. They were also more likely to be older and less likely to identify as another gender (compared with identifying as a woman) after adjusting for all other covariates (Table 1).

**Table 1.** Demographic Characteristics of Respondents

Characteristic	Respondents, No. (%)^a^	
	Full survey (N = 3802)	Analyzed subset (n = 2025)
Age, y		
18-19	7 (0.2)	<5
20-29	1073 (28.2)	396 (19.6)
30-39	1498 (39.4)	926 (45.7)
40-49	705 (18.5)	485 (24.0)
≥50	244 (6.4)	186 (9.2)
Race and ethnicity		
Asian	631 (16.6)	299 (14.8)
Black	212 (5.6)	151 (7.5)
Hispanic	52 (1.4)	23 (1.1)
Middle Eastern	117 (3.1)	55 (2.7)
Multiracial	45 (1.2)	26 (1.3)
Native American	<5	<5
White	2372 (62.4)	1303 (64.3)
Prefer to describe	268 (7.0)	134 (6.6)
Prefer not to answer	27 (0.7)	18 (0.9)
Gender		
Agender	6 (0.2)	<5
Gender fluid	5 (0.1)	<5
Gender queer	8 (0.2)	<5
Nonbinary	20 (0.5)	10 (0.5)
Man	322 (8.5)	149 (7.4)
Woman	3205 (84.3)	1860 (91.9)
Prefer to describe	<5	<5
Prefer not to answer	<5	<5
Sexual orientation		
Asexual	24 (0.6)	14 (0.7)
Bisexual	232 (6.1)	134 (6.6)
Demisexual	15 (0.4)	12 (0.6)
Fluid	11 (0.3)	6 (0.3)
Gay	58 (1.5)	22 (1.1)
Heterosexual	3022 (79.5)	1730 (85.4)
Lesbian	49 (1.3)	25 (1.2)
Pansexual	19 (0.5)	11 (0.5)
Queer	37 (1.0)	18 (0.9)
Questioning	28 (0.7)	16 (0.8)
Prefer to describe	14 (0.4)	6 (0.3)
Prefer not to answer	33 (0.9)	17 (0.8)
Title		
Medical student	1005 (26.4)	400 (19.8)
Resident	486 (12.8)	262 (12.9)
Fellow	210 (5.5)	132 (6.5)
Physician (independent practice)	1826 (48.0)	1200 (59.3)
Prefer to describe	37 (1.0)	16 (0.8)

^a^ Numbers do not add up to the total sample due to missing responses to specific items. We do not report exact values for items with fewer than 5 respondents. After adjusting for all other covariates in the logistic regression model, those in the analyzed subset were significantly older (*P* < .001); significantly more likely to be Black than White (*P* < .001); significantly more likely to identify as women vs other (nonbinary, agender, gender fluid, gender queer, or prefer to describe) genders (*P* = .04); significantly more likely to identify as bisexual vs heterosexual (*P* = .04); and significantly less likely to be a fellow than a physician in independent practice (*P* = .005). All pairwise contrasts for gender, sexual orientation, and race were considered, but after adjusting for multiple comparisons, no comparisons were significant for gender or sexual orientation. However, there were pairwise differences by race; those in the analyzed subset were significantly more likely to be Black than White (adjusted *P* < .001), Asian (adjusted *P* < .001), Middle Eastern (adjusted *P* < .001), or other races (Native American or prefer to describe) (adjusted *P* = .002).

In the analyzed subset of respondents, 1641 respondents (81%) answered question 1, 1786 (88%) answered question 2, and 1167 (58%) answered question 3. We describe findings across SEM levels: cultural, organizational, interpersonal, and individual (eFigure in Supplement 1). Themes describe results from all groups (students, residents, fellows, and physicians in independent practice) except when noted otherwise.

### Cultural

Respondents discussed obstacles at the cultural level of medicine (Table 2), including arduous training and discrimination.

**Table 2.** Example Quotes by Social-Ecological Level

Subtheme	Quotes (participant characteristics)^a^
**Cultural**	
Medicine’s culture of arduous training and competition that can be at odds with family building	“Prior to medical school, I was considering having (adopting and/or surrogate) children in residency. Now that I’ve experienced a sliver of medical training, there is no way that I would have kids during residency. Medical training is too involved, too time-consuming, and too demanding to be raising a child.” (man, gay, medical student, White) “No matter how supportive the training program, working 24 hour shifts and nights in pregnancy is dangerous and unacceptable and this is a part of the residency culture that we have deemed is acceptable.…Having children in residency is not sustainable and has pushed me to leave residency so that I can have the family size that I want.” (woman, heterosexual, resident, White) “I had my first child during 1st year of residency after a year of fertility problems. I was grateful for the pregnancy, but my intern year was brutal- long hours, 1:4 call schedule and no sympathy for the pregnant intern.” (woman, demisexual, physician, Black)^b^ “This has led me to be a single 34 year old woman who exists in the intersection of multiple identities, partnerless and childless despite my deepest desire to have my own family. After prolonged training, I now regret pursuing a surgical career. If I had known that this was part of the cost, I would not have entered this field.” (woman, heterosexual, fellow, Asian) “It made me realize how much of an impact the stress of becoming a physician wears on you. I can remember sitting in a shelf exam and starting to bleed. I was losing my baby but determined to finish my shelf exam. I was devastated. I promised myself I would never let the pressures of my career affect my ability to have kids. I was also very stressed during both pregnancies (one in medical school and one in residency). I wish I could have enjoyed them more.” (woman, bisexual, resident, Black)
Discrimination against pregnant individuals and those building families	“The minute I disclosed my pregnancy, I was no longer seen as serious, and felt that I was not on the same playing field as my mostly male peers. I then lost my fellowship as I was not ready to return to work as quickly as planned due to lack of childcare and support.” (woman, heterosexual, physician, multiracial) “When I tried to return to my career by doing a fellowship in my subspecialty again after leaving to have a child I became known as the one with the toddler, and some staff openly described me to others as having a ‘sad story.’ The reactions varied dramatically, and those from other women were oftentimes the harshest, seeming to somehow place a blame on me for having gotten myself into a situation in which I was in.” (woman, heterosexual, physician, Asian) “There is too much pressure on women to have children and to have them at a certain age, which obviously conflicts with the timeline for training. I chose not to have children, and am very likely to continue down the same path.” (woman, heterosexual, physician, White) “I had a miscarriage during rounds as a fellow in a hospital bathroom. There was so much stigma around being pregnant that I went back to round. I only told my mom. I was so afraid my program would find out about my second pregnancy.” (woman, heterosexual, physician, White) “I was told at my academic position that young women were less likely to get promotions because they were seen as a liability and unlikely to be reliable during/after pregnancy. I miss academics but do not miss this culture.” (woman, bisexual, physician, White)
**Organizational**	
Need for support for people of all genders and sexual orientations	“For gay trainees hoping to use surrogacy, it is a long and isolating process. Being gay, you already have to do things so much differently, and colleagues either don’t know or don’t think about what you may be going through throughout the surrogacy process.” (man, gay, physician, Asian) “My wife and I are both 40—we feel too old to conceive and also too old (and too gay) to be chosen as adoptive parents for an infant via private adoption. I wish I had thought about freezing my eggs early on during my training. I think it would have made it more of a natural step to become pregnant (even if later in life) or to have a surrogate. It’s just not something I ever discussed or heard about (particularly for lesbian women).” (woman, lesbian, physician, White) “It is unclear the support offered to same sex couples, in particular gay men, with respect to paternity leave. Not only that, there is a mental/social barrier to overcome as a gay man in medicine wanting to take leave to start a family. Also, depending on the location of the program, I am not sure I would even feel comfortable telling some attendings, PDs, chief residents, co-residents that I am gay; let alone request paternity leave in a same-sex relationship.” (man, gay, medical student, White) “I would have also invested in trying to change the laws around what insurance companies cover so that gay men aren’t excluded from having biological children. Many of my straight friends waited to have children till later due to their training and they were deemed infertile and their insurance covered IVF treatments. However, my husband and I, although we too couldn’t conceive together, had to spend over 200k to do surrogacy.” (man, gay, physician, White)
Lack of institutional and financial support for the range of family-building routes	“At the start of our journey, we were told to expect up to $200,000 worth of costs for a single pregnancy. Now that we have gone through two miscarriages, we’ve already spent $100,000 and now have to start the process of finding a new surrogate, labs, travel, genetic counseling, lawyers, surrogate insurance and IVF costs all over again. This is on top of the $500,000 in student loans we graduated with as a dual physician household. It feels so miserable knowing that you’ve committed so much time and sacrifice to medicine, and yet have to pay all medical costs out of pocket for surrogacy with zero assistance from insurance or ability to deduct any of the costs.” (man, gay, physician, Asian) “All of my money went towards medical school admissions/matriculation and now residency applications/matriculation. Hard to raise children when my career choice is so cost prohibitive.” (woman, bisexual, resident, Black) “Childcare is a big limitation. For residency, I was not in my home state and not familiar with the resources. I also saw others struggle due to lack of program and social support. Our hours are long and often not conducive to traditional daycare hours of 8-4 or 9-3.” (woman, heterosexual, unspecified career stage, Black) “I think there should be more discussion about pregnancy complications and how long it really takes to recover after childbirth. I got preeclampsia so had to be induced a few weeks early, but much more disruptive things can happen. And personally I can’t imagine having to go back to work after 2, 4, 6, or honestly even 8 weeks post-partum after my first pregnancy. It still hurt to pee, sit, and walk at 4 weeks, even though I didn’t have an exceptionally bad/unusual recovery in the scheme of things. I wish it was more feasible and even expected for residents and physicians out of training to take a more reasonable length of maternity leave, rather than having to return to work while your body is still in the acute phase of recovery.” (woman, heterosexual, medical student, multiracial)
Lack of transparency regarding institutional policies	“No one is talking about family planning while in medical school. I am applying for residencies where I plan to start a family during my training but have no idea which programs are transparent and supportive for starting families.” (woman, heterosexual, medical student, White) “I feel that my main reason for putting off having a family until now was the uncertainty of how my school/training would accommodate my having a child. If I had known or if there was some standardized policy that all training institutions had to abide by, that would have helped me make the decision sooner.” (woman, questioning, medical student, Asian)^c^ “The lack of clear policies and guidelines about maternal leave for trainees (including fellows) adds unnecessary stress. Policies should be described during residency and fellowship interviews. Trainees should not be forced to inquire about them. We should also allow paid time off for miscarriages and for pursuing IVF.” (woman, heterosexual, fellow, White) “I actively search for residency programs that listed on site child care or subsidized child care but not many have the options. I noticed a few programs have listed somewhere on website or contract about employee benefits subsidizing cost of adoption and IVF. So I wish more people know about it and more programs would list them publicly.” (woman, questioning, medical student, Asian)^c^
**Interpersonal**	
Social support from partner, family, and others	“I would not have chosen to have kids early in my training if I had not had an incredibly supportive and equitable partner. Having a partner committed to parenting equally was incredibly influential to my decision as has my success in training as a parent.” (woman, heterosexual, medical student, White) “[I should have] taken a residency position close enough to where my mother lives so she could help with emergency and other childcare. Having two children in a two-resident household without family support was very taxing mentally, emotionally, and financially.” (woman, bisexual, resident, White) “It [seems] impossible with both my partner and I being physicians. I worry we will have a child for it just to be hauled off to daycare.” (woman, heterosexual, resident, Hispanic) “Consider training close to where you have family or loved ones who can help with child rearing. It takes a village to raise a child, particularly when you’re in training.” (woman, heterosexual, resident, multiracial)
Relationship with coworkers	“Due to my training program handling maternity leave abysmally (including making it seem to my co-residents that I decided on a long leave at the last minute, causing people to have to cover during vacation/light rotations, even though I had actually scheduled several months before delivery), many of my co-residents developed negative feelings toward both me specifically and the idea of women having children during training more generally.” (woman, heterosexual, physician, Black) “Put more pressure on my Co-residents not to have babies and my program to have better plans for making people make call even—as the only resident in my program that didn’t have kids I took more call than everyone in my class combined and at the end of my senior year nearly committed suicide because of the stress and sleep deprivation.” (woman, asexual, physician, White) “I would have started a little earlier and taken a longer maternity leave—the program could have handled it but I was too nervous to be a burden on my peers (that would have had to cover my inpatient rotations).” (woman, heterosexual, unspecified career stage, Asian) “As a female who has chosen to not have children, I feel like I get unfairly asked to cover many less desirable shifts for those that do have children, as if I don’t have loved ones I want to spend time with. Hence, my choice to not procreate means that I get stuck with worse hours and more holiday coverage even though academia claims to be fair to all housestaff.” (woman, pansexual, physician, Asian)
**Individual**	
Impact of socioeconomic status	“I’m very aware of the extent to which medicine is not actually a meritocracy and that the best thing I could have done for my career path was to be born to different, richer, better educated lighter skinned parents.” (gender queer, bisexual, resident, Black) “I think if someone is strongly considering children at some point during their training they probably have the financial or familial support to let themselves want children and could probably handle having a child, albeit still challenging. Those not in a situation to be able to support children financially or via family supports are likely suppressing their desire to have children and not strongly considering children because it simply isn’t feasible for them at that point in their lives.” (woman, heterosexual, medical student, White) “We could not financially afford a third child when our family was ready for a third child, and now our two children are far removed from the baby stage so my husband does not want to ‘go backwards.’ So even though we will be financially stable as attendings and could certainly afford three or more children, having children during residency has limited the number of children that our particular family can have.” (woman, bisexual, resident, White) “I never knew the costs for surrogacy as a same sex couple until I was in my early 30s and realized how difficult it is to save that amount of money while still paying student debt. I also realized that the field of medicine I chose offers one of the lowest salaries, which has made it difficult to balance starting a family and other debt.” (man, gay, physician, Middle Eastern)
Additional individual factors, eg, military status, immigrating from other countries	“Immigrating from a foreign medical system to a competitive specialty requiring additional years of academic investment in pre-residency training to qualify for an eventual residency spot as well as the financial and time pressures of credentialing in the US and preparing for the corresponding licensure examinations has been the one part of my career with the largest impact on my/our family planning decisions.” (man, heterosexual, pre-residency research fellow, Asian) “Being a foreign medical graduate, my husband and I were separated for years during training and first 3 of job due to visa issues. This has happened to many of IMG colleagues. If we didn’t have this separation, perhaps would have been in a different situation.” (woman, heterosexual, physician, Asian) “I am a military resident, which complicates things even further. EM is a highly deployable specialty, likely to face 2-3 deployments during a 4 year active duty attending period. I will be 32-36 for those years. If I have kids now, while still in training, they will be 18mo-3 years old for those potential deployments. It’s hard to imagine leaving them for 4-6mo at a time. But at the same time, I can’t and won’t put off having a family because of those potential interruptions.” (woman, heterosexual, resident, White) “The Army has actually made it easier. I was fortunate to be located at a hospital with one of their REI programs. The military healthcare system covered about half our costs. I had no issues arranging work around my appointments.” (woman, heterosexual, physician, White)

Abbreviations: EM, emergency medicine; IMG, international medical graduate; IVF, in vitro fertilization; PD, program director; REI, reproductive endocrinology and infertility.

^a^ Responses shared verbatim without correction of grammatical errors.

^b^ Physician indicates physician in independent practice.^.^

^c^ Comments from 2 different participants.

#### Medicine’s Culture of Training

The demanding nature of medicine, especially in residency, involving long shifts and a competitive environment, presented barriers to family building. It is particularly challenging for those who are pregnant, undergo in vitro fertilization (IVF) treatments, and build families through surrogacy, fostering, or adoption. Some respondents described how family building negatively impacted their career, including via reputational damage, career advancement delays, and negative relationships with coworkers and leadership. Some stated they may have chosen a different career or specialty if they knew how challenging family building would be. Respondents described particular work environments (eg, procedural specialties, academic medicine) as less family friendly, leading some to choose alternative career paths than otherwise desired. Respondents discussed that many medical schools do not facilitate conversations about family building: “[there was a] lack of formal, or even informal, dialogues from my institution regarding family planning.”

#### Discrimination Against Pregnant Individuals and Those Building Families

Pregnant individuals and individuals building families reported discrimination from their programs, including being less likely to be promoted. Pregnant physicians—particularly those who have completed training—shared experiences of discrimination: “[faculty] have made it painfully clear they would look down on and resent any female colleague who got pregnant.” This discrimination can manifest in supervisors and peers discouraging individuals from having children or individuals being “advised casually” to delay children until later in their careers, particularly after training. Some respondents reported that pressure to delay resulted in infertility and fewer children than otherwise desired. A small number of respondents reported being surprised by “overwhelmingly positive and supportive” responses from their colleagues during family building.

Although some respondents suggested times that were easier for them to have children than others, many individuals endorsed the belief that “there is no perfect time.” Respondents discussed how it is not necessarily easier to build families after residency and, thus, encouraged others to not give into pressure to delay. While many respondents expressed that their decision to have children was independent of their career, the perspective of medicine being incompatible with family building resulted in some respondents forgoing children to maintain their career path. Respondents commented on the tension between the societal pressure to have children and the culture of medicine, which produced barriers for family building.

#### Cultural-Level Recommendations

Respondents recommended addressing cultural-level challenges, including by implementing family-building curricula for students, modifying schedules for those building families, and creating humane schedules for all physicians and students (Table 3). Establishing diverse leadership with individuals of all genders and sexual orientations was recommended to reduce discrimination resulting in pressure to delay family building.

**Table 3.** Participant Recommendations for Improving Support for Family Building Across Social-Ecological Levels

Social-ecological level	Recommendations
Cultural	Modify schedules for those building families (particularly considering night shifts and longer shifts) and create more humane schedules for all physicians and medical students. Establish diverse medical leadership with individuals of all genders and sexual orientations to promote a more positive working environment and reduce discrimination that results in pressure to delay family building.
Organizational	Improve policies for all individuals desiring to build families—regardless of gender, sexual orientation, or ability to become pregnant—including insurance coverage for cryopreservation, ART, adoption, and gestational carriers. Provide financial assistance for physicians and medical students wishing to pursue ART. Build on current ACGME recommendations by extending the duration of parental leave and offering it to all parents (not just pregnant parents) and improve leave policies for people who have experienced miscarriages, abortion, postpartum depression, ART, and other events. Consider competency-based training (instead of time-based). Provide reentry funding options for physician scientists after parental leave. Increase protection for lactation. Improve access to onsite, 24/7 childcare. Publicly list program policies and proactively communicate these to interviewing applicants to create more transparency and accountability. Increase options for part-time work. Increase support for physicians and medical students who have experienced domestic, family, and sexual violence as well as mental health challenges, including postpartum depression.
Interpersonal	Hire covering clinicians for physicians on leave to alleviate stressors on colleagues and decrease resentment of those taking leave.
Individual	Organizational-level recommendations noted previously, such as greater support via insurance, parental leave, childcare, and so on, could alleviate some of the challenges at the individual level.

Abbreviations: ACGME, Accreditation Council for Graduate Medical Education; ART, assisted reproductive technologies.

### Organizational

Challenges at the organizational level (Table 2) included the negative impact of heteronormative policies, lack of institutional support, and lack of transparency.

#### Policies Across Sexual Orientations and Gender Identities

Respondents reported unclear or lacking policies surrounding family building for LGBTQIA+ physicians, including lack of insurance coverage. Respondents who identified as LGBTQIA+ described a lack of resources specific to their needs, experiences of discrimination, a lack of conversations about family building, and the high cost of family-building routes.

#### Policies Across the Range of Family-Building Routes

For individuals building families through surrogacy, ART, and other high-cost routes, a lack of institutional financial support (eg, insurance coverage) impaired the ability to have children or preserve the ability to do so: “residency salaries alone and the insurance coverage they give us is not enough for me to afford freezing my eggs.” The high cost of medical training made it challenging for those wanting to build families, particularly during training periods with significant incurred debt and low salaries. Individuals undergoing IVF treatments may not be given needed institutional support to cover shifts for appointments or for taking “medications and injections while on shift.” Respondents described parental leave as short as 4 weeks for pregnant individuals and unclear or nonexistent parental leave policies for other new parents. Lack of institutional support for childcare also contributed to respondents’ family-building decisions and career outcomes. The residency match process further contributed to these challenges by placing some residents far from support networks, leading to problems with childcare access and increased stress or desires to not have children.

Respondents described inadequate support surrounding pregnancy outcomes and complications (including miscarriage and abortion): “[The] chairman made me do prenatal clinic the day after I miscarried.” Some respondents suggested that the physical, emotional, and psychological demands of a medical career may be related to adverse outcomes such as miscarriage and infertility. Particular concerns around radiation exposure and its impact on pregnancy were reported by physicians in specialties such as radiology.

#### Participant Knowledge of Institutional Policies

Respondents expressed frustration with a “lack of clear policies and guidelines” on family building. Some, especially students, reported that finding policies on parental leave, financial support, and childcare, among other factors, was challenging at many medical schools and health systems.

#### Organizational-Level Recommendations

Recommendations at the organizational level included the need for programs to improve policies for all individuals desiring to build families—regardless of gender, sexual orientation, or ability to become pregnant—including insurance coverage for cryopreservation, ART, adoption, and gestational carriers (Table 3). Additional recommendations included: providing longer parental leave for all parents; considering competency-based training (instead of time-based); increasing protection for lactation; improving access to onsite, 24/7 childcare, and publicly listing policies and proactively communicating these to interviewing students and physicians.

### Interpersonal

Respondents shared the importance of social support when building families. They also expressed the desire to maintain positive relationships with coworkers (Table 2).

#### Social Support

Support from one’s social network largely facilitated positive family-building experiences. Some respondents reported living close to members of their social network enabled them to overcome barriers to family building, including childcare. Having a “supportive and equitable partner” also allowed some respondents to feel they had more control over the timing of children, including planning to have children during residency. Some respondents indicated that a “dual-physician household” may contribute to additional barriers; for instance, in terms of “limitations of partner’s time and ability to support as well as the possibility of being long-distance for training.” Some respondents also noted that the demands of training led to difficulty in finding a partner, impacting family building.

#### Relationships With Coworkers

Respondents without children reported added stress and “significant personal cost” to their well-being when overextended in institutions without adequate coverage for physicians on parental leave: “My work burden increased to accommodate my coresidents’ maternity leave, and while I fully support their decision to have kids during residency, there’s still a penalty that others pay.” Individuals taking parental leave reported “negative feelings” from their coworkers because they had children.

#### Interpersonal-Level Recommendations

Hiring covering clinicians for physicians on leave could alleviate stressors experienced by individuals without children and those having children (Table 3).

### Individual

Individual factors, such as financial resources, also impacted respondents’ family-building journeys (Table 2).

#### Socioeconomic Status

Although physicians are, on the whole, a privileged group, respondents discussed how socioeconomic status was a factor in family building. Respondents reported that access to financial resources facilitated family building, especially when there was a lack of institutional support. They also reported that individuals without personal resources to finance ART for cryopreservation or pregnancy were less likely to build families as they desired.

#### Additional Individual Factors

Further individual factors impacting family building included immigration status, which may require “additional years of academic investment in pre-residency training” (Table 2). Some respondents noted military status negatively impacted family building through deployment and separation from partners; several respondents described benefitting from the military’s health care system.

#### Individual-Level Recommendations

Organizational-level recommendations noted previously (eg, greater financial support, parental leave, childcare) could alleviate some challenges at the individual level (Table 3).

## Discussion

Across different levels of medical training and practice, the stories from more than 2000 respondents in this study depict a harsh reality in which the medical profession creates and sustains barriers to family building. These barriers exist at every level of the SEM (cultural, organizational, interpersonal, and individual). Our findings point to cultural and organizational levels producing the most significant barriers to family building for physicians and students. LGBTQIA+ physicians and students experience further barriers to family building, facing additional discrimination and lack of clear and equitable policies. Although those in other occupations may face similar barriers, the extended duration of training (and concomitant low compensation) in medicine combined with lack of support for family building create unique challenges.^1^

Our data suggest arduous training and a competitive environment produce significant pressure on physicians and students to avoid family building until after completing training. Many pregnant individuals and those with children—particularly women and LGBTQIA+ individuals—reported experiencing discrimination, a finding also demonstrated in a survey study of more than 1000 women oncologists.^29^ For many respondents, discrimination and the high-stress environment of medicine ultimately resulted in smaller-than-desired family sizes or the inability to have children. This is consistent with a prior survey of 1021 women surgeons showing they had fewer children, had their first pregnancies later in life, and reported more fertility issues than the general US population.^30^ These barriers caused some physicians and students to change their medical specialty or leave medicine altogether. Other studies similarly found that many women physicians considered changing their specialty or career due to experiences with discrimination and family building.^7,31^ Respondents’ suggestion that medical schools should implement curricula on family building is consistent with prior literature: a survey study of 175 medical students demonstrated a desire for strengthening family-building education.^32^

Barriers at the organizational level included a lack of transparency surrounding institutional policies and a lack of financial support for all family-building routes. When people grow their families despite these barriers, they face inconsistent, inadequate, and often unclear policies regarding parental leave, coverage for fertility treatment, childcare, and support for pregnancy loss. In a study of 15 graduate medical education–sponsoring programs, the mean designated parental leave was 5.7 weeks for birthing parents and 3.9 weeks for nonbirthing parents.^6^ Many appreciated the recently updated guidelines from the Accreditation Council for Graduate Medical Education and the American Board of Medical Specialties, which mandate at least 6 weeks of parental leave, although they fall short of the 12 weeks recommended by the American Academy of Pediatrics.^33,34,35^ The chief rotation requirement of the American Board of Surgery may restrict surgery residents from obtaining 6 weeks of leave without delaying their graduation.^36^ Recommendations to improve parental leave and increase clinical coverage for parents on leave are consistent with prior studies and commentaries.^37,38,39,40,41^ Although not mentioned by respondents in this study, other forms of leave, such as for caregiving, would likely also be helpful.

To our knowledge, this study is one of the first on family building for physicians and students that includes the experiences of individuals of all gender identities and sexual orientations. Respondents discussed unique challenges related to the lack of insurance coverage, financial resources for ART, and parental leave for all parents, not only pregnant people. In the study of 15 graduate medical education institutions,^6^ less than half of these institutions had paid parental leave policies for nonbirthing parents, and only 6 used inclusive language for LGBTQIA+ individuals and adoptive parents. Improving insurance coverage for cryopreservation, ART, adoption, gestational carriers, and other pathways can support the family-building experiences of all physicians and students building families, not only cisgender, heterosexual individuals. Continued research on family building for physicians and students must include an intersectional lens to ensure the equitable implementation of recommended practices.

### Limitations

This study has limitations, including the inability to calculate a response rate due to the recruitment strategy. There is also the potential for selection bias due to convenience sampling using social media or due to those desiring to have children or those with family-building challenges potentially being more likely to respond. We observed an imbalance of respondents’ demographic characteristics in the study, with most identifying as White, as heterosexual, and as women. Furthermore, future research should seek to better understand and quantify the desires of those who do not want or have children, who were not well represented in our data.

## Conclusions

In this qualitative study of physicians and medical students, we identified major barriers in family building. Cultural, organizational, interpersonal, and individual factors make it difficult for physicians and students to build the families they desire due to their dedication to improving the health of their communities. This study presents recommendations that hospitals, clinics, and medical schools should use to promote their physicians’ and students’ physical, emotional, and reproductive well-being.

## Notes

Supplement 1. **eFigure. **Barriers and Facilitators to Family Building for Physicians and Medical Students Organized by Socioecological Model Levels

Supplement 2.
Data Sharing Statement
