# Supplementary material for: Barriers to Family Building Among Physicians and Medical Students
Source: JAMA Netw Open. 2023 Dec 28;6(12):e2349937. doi: 10.1001/jamanetworkopen.2023.49937 (PMC10755597; doi:10.1001/jamanetworkopen.2023.49937)
Supplement: Supplement 1. — eFigure. Barriers and Facilitators to Family Building for Physicians and Medical Students Organized by Socioecological Model Levels [file jamanetwopen-e2349937-s001.pdf]

Supplemental Online Content

King Z, Zhang Q, Liang JW, et al. Barriers to family building among physicians and medical students. *JAMA Netw Open*. 2023;6(12):e2349937. doi:10.1001/jamanetworkopen.2023.49937

eFigure. Barriers and Facilitators to Family Building for Physicians and Medical Students Organized by Socioecological Model Levels

This supplemental material has been provided by the authors to give readers additional information about their work.

© 2023 King Z et al. *JAMA Network Open.*

eFigure. Barriers and Facilitators to Family Building for Physicians and Medical Students Organized by Socioecological Model Levels

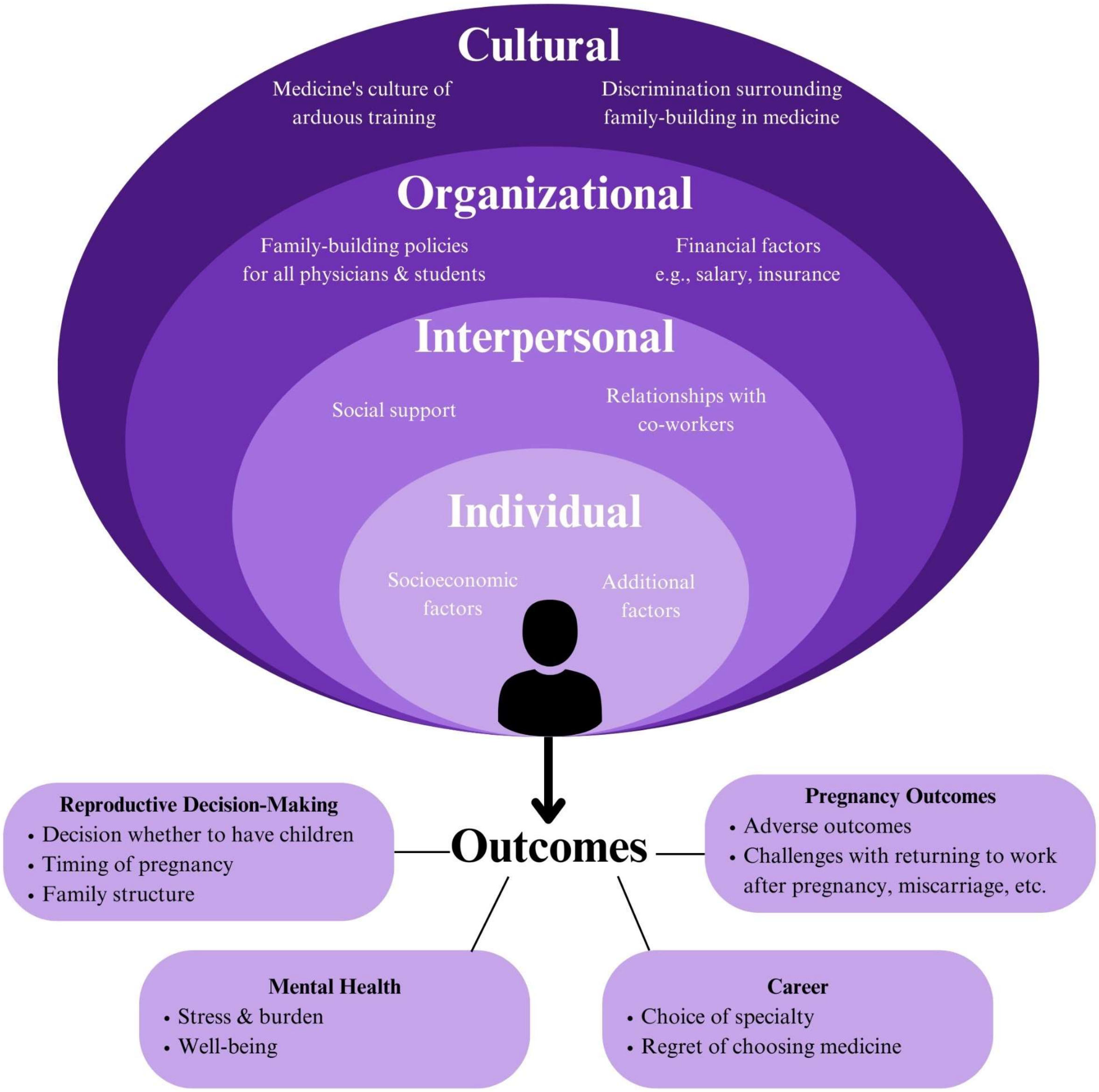

© 2023 King Z et al. *JAMA Network Open.*
