# Supplementary material for: Barriers to Family Building Among Physicians and Medical Students
Source: JAMA Netw Open. 2023 Dec 28;6(12):e2349937. doi: 10.1001/jamanetworkopen.2023.49937 (PMC10755597; doi:10.1001/jamanetworkopen.2023.49937)
Supplement: Supplement 2. — Data Sharing Statement [file jamanetwopen-e2349937-s002.pdf]

Data Sharing Statement

King. Barriers to Family Building Among Physicians and Medical Students. *JAMA Netw Open*. Published December 28, 2023. doi:10.1001/jamanetworkopen.2023.49937

Data
Data available: No

Additional Information
Explanation for why data not available: We will make the deidentified data available upon request but will not be posting it publicly.
